# The relationship between P2X4 and P2X7: a physiologically important interaction?

**DOI:** 10.3389/fphys.2013.00216

**Published:** 2013-08-15

**Authors:** Eilidh Craigie, Rebecca E. Birch, Robert J. Unwin, Scott S. Wildman

**Affiliations:** ^1^Centre for Nephrology, UCL Medical School, University College LondonLondon, UK; ^2^Urinary System Physiology Unit, Medway School of Pharmacy, Universities of Kent and Greenwich at MedwayKent, UK

**Keywords:** purinergic, kidney, P2X4, P2X7, receptor

## Abstract

Purinergic signaling within the kidney is becoming an important focus in the study of renal health and disease. The effectors of ATP signaling, the P2Y and P2X receptors, are expressed to varying extents in and along the nephron. There are many studies demonstrating the importance of the P2Y_2_ receptor on kidney function, and other P2 receptors are now emerging as participants in renal regulation. The P2X4 receptor has been linked to epithelial sodium transport in the nephron and expression levels of the P2X7 receptor are up-regulated in certain pathophysiological states. P2X7 antagonism has been shown to ameliorate rodent models of DOCA salt-induced hypertension and P2X4 null mice are hypertensive. Interestingly, polymorphisms in the genetic loci of P2X4 and P2X7 have been linked to blood pressure variation in human studies. In addition to the increasing evidence linking these two P2X receptors to renal function and health, a number of studies link the two receptors in terms of physical associations between their subunits, demonstrated both *in vitro* and *in vivo*. This review will analyze the current literature regarding interactions between P2X4 and P2X7 and assess the potential impact of these with respect to renal function.

## Introduction

ATP is an important extracellular signaling molecule that is the principal agonist for two different types of purinergic receptors: P2Y (G protein-coupled receptors) and P2X (non-selective ion channels). Eight different subtypes have been identified for P2Y (1,2,4,6,11–14) and seven for P2X (1–7), and both P2Y and P2X receptors are found in virtually all mammalian tissues and participate in many different physiological processes [for recent reviews refer to (von Kugelgen and Harden, 2011) and (Surprenant and North, 2009)].

Purinergic signaling within the kidney is emerging as an important focus in the study of renal health and disease, and P2X and P2Y receptors are expressed to varying extents along the nephron; some are more ubiquitously expressed than others, and some alter their expression levels depending on the physiological and pathophysiological state of the kidney (for recent reviews covering P2 receptors in the kidney refer to (Vallon, 2008; Praetorius and Leipziger, 2010; Booth et al., 2012). There are numerous studies comprehensively demonstrating the importance of the P2Y_2_ receptor on kidney function (Vallon and Rieg, 2011), but P2X receptors are also beginning to emerge as important participants in kidney homeostasis (Bailey et al., 2012).

The recent success in obtaining the first x-ray crystallography structure of a P2X receptor (Kawate et al., 2009) verified previous evidence that functional P2X receptors are composed of three subunits (Nicke et al., 1998; Barrera et al., 2005). All subunits (except for P2X6) assemble to form homotrimeric receptors, and some can be assembled in various configurations to form heterotrimeric P2X receptors (Torres et al., 1999; North, 2002) which display unique pharmacological profiles compared with homotrimers (Lewis et al., 1995; King et al., 2000). P2X7 is distinctive, as in the extensive heterotrimer study by Torres et al. it was the only P2X subunit found not to interact with others to form heterotrimers (Torres et al., 1999); however subsequent studies, as discussed in detail below, have suggested that this subunit may in fact be capable of interacting with other subunits. The amino acid (aa) sequence of P2X7 is considerably longer than all other P2X subunits due to an extended cytoplasmic tail (595 aa vs. 388–471 aa for P2X1-6) and this is reflected in a larger protein mass (P2X1-6 = 43.4–51.7 KDa, P2X7 = 68.6 KDa). The P2X7 receptor also possesses a significantly lower affinity for ATP than all other P2X receptors [EC_50_ = 1–10 μM for P2X1-6 vs. >100 μM for P2X7 (North and Surprenant, 2000)].

The P2X subunit with which P2X7 shares most similarity is P2X4; they are the most closely related of all P2X in terms of aa sequence (48.6% similarity for human sequence and 49.8% for rat), their chromosomal location (only 24 Kb apart on human chromosome 12), and their overlap in tissue distribution, particularly in immune, endothelial, and epithelial cells (Soto et al., 1996; Murrell-Lagnado and Qureshi, 2008). Given the similarities between these two receptors, researchers have sought to identify if there is a physical and functional interaction between them. Recent reports suggest a close interaction between P2X4 and P2X7, although whether subunits assemble to form functional heterotrimers or interact as homotrimeric receptors to form cooperative receptor complexes is still unclear. We have reviewed the recent literature on the emerging relationship between P2X4 and P2X7, and focus on the potential role of these two receptors in kidney function.

## P2X4 and P2X7 interactions

Evidence from an alveolar cell line suggests that P2X4 and P2X7 receptors can influence the expression of one another; P2X4 receptor expression is up-regulated when P2X7 receptor expression is knocked down, and P2X7 receptor expression is increased when P2X4 receptor expression is decreased (Weinhold et al., 2010). The authors also report alterations in the cellular localization of both receptors during their altered expression profiles, with plasma membrane expression becoming more pronounced in both instances.

**Figure 1:**
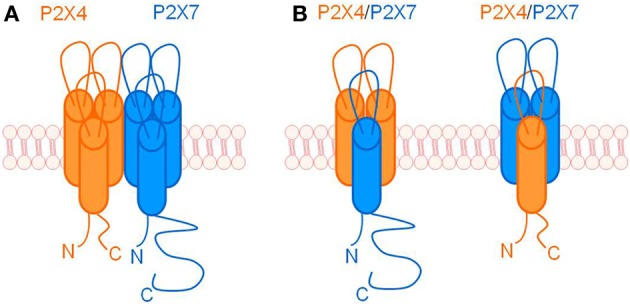
**Schematic representation of ways in which P2X4 and P2X7 may interact**. **(A)** Individual P2X4 and P2X7 homotrimers physically associating with one another, possibly via C-terminal tail interactions or intermediate scaffolding proteins. **(B)** P2X4 and P2X7 subunits forming heterotrimeric receptors.

Prolonged activation of some P2X receptors can induce formation of non-selective pores that are permeable to large molecules, including fluorescent dyes (North, 2002; Pelegrin, 2011). It was previously believed that channel to pore formation was a unique property of the P2X7 receptor, however, many studies now provide evidence that other subunits can also participate in pore formation e.g., P2X2 and P2X4 (Virginio et al., 1999), P2X4, P2X2 and P2X2/3 heterotrimeric complexes (Khakh et al., 1999), and P2X2/5 heterotrimeric complexes (Compan et al., 2012). The potential for interactions between P2X4 and P2X7 to influence pore formation was explored using a HEK-293 heterologous expression system where co-expression induced an altered response to ATP and fluorescent dye uptake compared with expression of P2X7 alone (Casas-Pruneda et al., 2009). This study provides evidence supporting a functional interaction between P2X4 and P2X7, although it was unable to ascertain the precise mechanism of this interaction; see Figure 1 for a schematic representation of possible mechanisms.

The P2X_cilia_ channel receptor is an unidentified P2X receptor expressed in airway ciliated cells; unidentified in that its pharmacology differs from all other previously described P2X homo- or heterotrimers (Korngreen et al., 1998; Ma et al., 1999). Ma et al. presented evidence to suggest that P2X_cilia_ may consist of both P2X4 and P2X7 subunits (Ma et al., 2006), demonstrating that the P2X_cilia_ receptor shares properties in common with P2X4 and P2X7 homotrimers and showing positive immuno-staining for P2X4 in the basal cilia where P2X_cilia_ is expressed. However, the immuno-staining for P2X7 at this location was incomplete and the study was inconclusive as to whether the P2X_cilia_ receptor channel is a P2X4 and P2X7 heterotrimer. Guo et al. subsequently proposed that P2X4 and P2X7 subunits can form functional heterotrimers (Guo et al., 2007). Using a HEK-293 cell heterologous expression system they demonstrated that the surface expression of P2X4 increased more than 2-fold when it was co-expressed with P2X7 and that the subunits physically interacted using immunoprecipitation methods; they were also able to demonstrate that this interaction occurs in native tissue, specifically macrophages. In addition, studies using non-functional mutant P2X4 subunits in the *Xenopus* oocyte heterologous expression system revealed that when they were co-expressed alongside normal P2X7 subunits, the pharmacology of P2X7 receptors was altered. These demonstrations of both physical and functional interactions between P2X4 and P2X7 subunits prompted the authors to suggest that this was evidence for a heterotrimeric assembly.

Subsequent analyses of endogenous P2X4 and P2X7 interactions have indicated a preference for the formation of separate homotrimers that have a close physical interaction, rather than heterotrimers (Nicke, 2008; Boumechache et al., 2009). Chemical cross-linking analysis was used by Antonio et al. in a transfected cell line to show that although P2X4 and P2X7 are in close proximity to each other, no heterotrimeric receptors were formed (Antonio et al., 2011). In addition, atomic force microscopy imaging confirmed that receptor dimers were present and, although the identification of specific homotrimers was not possible, this, along with the results of the close proximity assay, supports the hypothesis that P2X4 and P2X7 homotrimers interact. However, what do these interactions mean for the function of P2X4 and P2X7? A study by Casas-Pruneda et al. shows that P2X4 and P2X7 receptors functionally interact in both a heterologous system (HEK-293 cells) and native epithelia (mouse parotid acinar cells), and that they work together to produce an ATP-activated inward current with functional and pharmacological characteristics that are distinct from either individual homotrimer (Casas-Pruneda et al., 2009). Functional interactions between P2X4 and P2X7 have also been demonstrated in mouse immune cells; normal P2X7 receptor-dependent functions (e.g., P2X7-mediated cell death and release of inflammatory signals) are altered when P2X4 expression levels are reduced (Kawano et al., 2012a,b; Sakaki et al., 2013). The current literature suggests that P2X4 and P2X7 are involved in functionally relevant interactions, both in native and transfected systems, and that the most likely explanation for their close relationship is that they are behaving in a cooperative manner as separate, but physically interacting, homotrimeric receptors.

## Purinergic signaling in the kidney

All of the key elements of the purinergic signaling system are found within the kidney, including P2 receptors, adenosine receptors, and a range of different ectonucleotidases [for recent review of renal ectonucleotidases see (Shirley et al., 2009)]. In addition, renal tubular cells are capable of releasing nucleotides in response to stimuli such as mechanical stimulation (e.g., stretch, increased flow rate, and osmotic swelling), local acidosis, hypoxia, and various hormones (e.g., vasopressin and aldosterone) (Vekaria et al., 2006; Odgaard et al., 2009). This accumulated evidence suggests a role for nucleotides in regulating renal function.

Functionally, P2 receptors have been shown to have important regulatory effects upon the kidney e.g., P2X1 receptor expression in the afferent arteriole of the glomerulus has been established to have a role in renal blood flow autoregulation (Inscho et al., 2004). With regards to epithelial transport, there are many studies demonstrating the importance of the P2Y_2_ receptor in the kidney and physiological studies using the P2Y_2_ null mouse model (P2Y^−/−^_2_) have been instrumental in these [for recent overview see (Vallon and Rieg, 2011)]. In the distal nephron P2Y_2_ receptor activation has been shown to mediate the inhibitory effect of both dietary sodium and aldosterone escape upon epithelial sodium channel (ENaC)-mediated sodium reabsorption (Pochynyuk et al., 2010; Stockand et al., 2010). Additionally, P2Y^−/−^_2_ mice have a reduced ability to down regulate ENaC activity in response to nucleotide signaling and this is thought to contribute toward their hypertensive phenotype (Pochynyuk et al., 2008). In the thick ascending limb (TAL), P2Y^−/−^_2_ mice have a greater expression of the sodium potassium chloride co-transporter type 2 (NKCC2) and increased furosemide-induced natriuresis, compared with control mice (Rieg et al., 2007; Zhang et al., 2011).

P2X4 and P2X7 are also expressed in the kidney, and genetically modified knockout mice exist for both, enabling researchers to investigate their involvement in the physiology and pathophysiology of tissues of expression, including the kidney. Some of these, along with *in vitro* studies investigating P2X4 and P2X7, will be discussed, with particular emphasis on renal function.

### Renal P2X4

P2X4 is expressed throughout the kidney (Schwiebert and Kishore, 2001; Turner et al., 2003), and many of the cell-based studies investigating renal P2X4 have focused on its potential to influence epithelial sodium transport. McCoy et al. used a mouse-derived collecting duct (CD) cell line to show that activation of apical P2X, as well as P2Y, receptors had inhibitory effects on ENaC activity; they showed that these cells express P2X4 mRNA (McCoy et al., 1999). This suggested a possible link between P2X receptor activation (tentatively P2X4) and ENaC activity. Following on from this, Gorelik et al. used a *Xenopus*-derived renal epithelial cell line to show that activation of unspecified P2 receptors at the basolateral membrane induced changes in cell structure that altered the surface of the apical membrane in a way that permitted ENaC to become more active (Gorelik et al., 2005). The researchers built on this using a series of P2 receptor agonists and antagonists to show that the effects were most likely P2X4-dependent, and suggested that apical stimulation of P2X receptors has an inhibitory effect up on ENaC, while basolateral stimulation is potentiating (Zhang et al., 2007). In an oocyte heterologous expression system we demonstrated that P2X4 activation was able to inhibit ENaC currents due to a decrease in ENaC surface expression, demonstrating direct apical regulation of ENaC by a P2X receptor (Wildman et al., 2005). Together, these studies suggested a possible role for P2X4 in renal sodium transport, in particular via ENaC, and that there may be opposing actions for apical vs. basolateral activation. This was subsequently corroborated in native tissue using micro-dissected rat CDs; CDs were split-open and whole-cell patch-clamp electrophysiology was performed on the accessible apical membrane (Wildman et al., 2008). Using specific P2 receptor agonists these experiments showed that a P2X receptor was able to inhibit ENaC activity when the sodium concentration of the experimental bathing fluid was 145 mM (standard conditions for these experiments). This P2X receptor was thought to be a P2X4/6 heterotrimer, given the localization of these two receptors, and taking in to consideration the earlier work of Torres and colleagues (Torres et al., 1999). However, in retrospect, there is the possibility of a P2X4 and P2X7 heterotrimer and/or homotrimer interaction. When the sodium concentration of the bathing solution was reduced to 50 mM [more representative of distal tubular fluid sodium concentration (Malnic et al., 1966)], we saw a shift from inhibition to potentiation of ENaC activity by P2X4 activation. We also showed robust expression of the P2X4 receptor at both the apical and basolateral membranes of rat CDs using immunofluorescence. These observations led us to suggest that P2X4 receptors may be capable of responding to the sodium concentration of distal tubular fluid to influence ENaC activity at a local level, and assist the kidneys in maintaining sodium balance.

Most studies published to date using the P2X4 null (P2X4^−/−^) mouse model have not concerned renal function, and have focused predominantly on the receptor's role in pain, inflammation, and synaptic signaling (Sim et al., 2006; Brone et al., 2007; Tsuda et al., 2009; Ulmann et al., 2010). Interestingly, P2X4^−/−^ mice are hypertensive and a study investigating this linked it to endothelial dysfunction and impaired vasodilation (Yamamoto et al., 2006). Renal function was not investigated in this study and so the contribution of lack of P2X4 in the kidney to hypertension is unknown; this possibility should not be discounted, since almost all forms of inheritable hyper- and hypotension identified in humans have evidence of abnormal renal sodium handling (Lifton et al., 2001). Moreover, in a recent series of isolated perfused tubule experiments, Marques et al. demonstrated that P2X4^−/−^ mice had a blunted response to ATP-mediated inhibition of sodium reabsorption in micro-dissected TALs (Marques et al., 2012). This shows a direct effect of P2X4 on epithelial sodium transport in native tissue, and indicates that these P2X4^−/−^ mice have enhanced renal sodium reabsorption which may contribute toward their hypertension.

### Renal P2X7

P2X7 is constitutively expressed in the majority of immune cells and receptor activation has broad pro-inflammatory effects. Consequently, the P2X7 null (P2X7^−/−^) mouse model has been extensively used to study inflammation in renal pathophysiology, and expression patterns of P2X7 have been mapped in models of inflammation. A major theme of these studies is that P2X7 expression is up-regulated in diseased/inflamed renal tissue, and that a lack of expression, such as in P2X7^−/−^ mice, offers varying degrees of protection. For example, the induction of unilateral ureteric obstruction is a widely used model of early inflammation and tubulointerstitial fibrosis with progressive kidney injury; this is attenuated in P2X7^−/−^ mice, which show less fibrosis, as well as reduced macrophage infiltration and expression of inflammatory cytokines (Goncalves et al., 2006). A key role for P2X7 in glomerulonephritis was also identified using rodent models of nephrotoxic nephritis in P2X7^−/−^ mice (Turner et al., 2007) and after pharmacological inhibition of P2X7 in rats (Taylor et al., 2009). Moreover, P2X7 expression is up-regulated in the glomerulus and tubular cells in human lupus nephritis, a condition in which glomerular inflammation is an important feature (Turner et al., 2007).

Some studies have found that the P2X7 receptor is expressed in renal epithelial cells (Schwiebert and Kishore, 2001; Hillman et al., 2004), although its function in non-immune cells is less clear. Under *in vitro* conditions, P2X7 receptors can mediate direct renal epithelial cell-fibroblast crosstalk following tubular damage; necrotic tubular cells have been shown to promote interstitial fibroblast cell death by a P2X7-dependent mechanism (Ponnusamy et al., 2011). P2X7 is also involved in polycystic kidney disease (PKD): in a mouse model of autosomal recessive PKD, P2X7 expression is up-regulated in CD cells as they undergo cystogenesis; in addition, P2X7 antagonism can reduce cyst number, but not size, in CD suspension cultures (Hillman et al., 2002, 2004). Contrary to this, P2X7 receptor blockade was shown to prevent cyst enlargement, but not frequency, in a zebrafish model of autosomal dominant PKD (Chang et al., 2011).

P2X7 expression in the kidney has also been linked to rodent models of hypertension, which is interesting, because an association study into human hypertension has shown a link between polymorphisms in the P2X4 and P2X7 gene region and blood pressure regulation (Palomino-Doza et al., 2008). In a renin over-expressing rat model of hypertension the expression of P2X7 is up-regulated in the glomerulus (Vonend et al., 2004). Additionally, hypertension and renal injury are attenuated in P2X7^−/−^ mice with DOCA salt-induced hypertension, and expression of the P2X7 receptor is increased in DOCA salt-treated mice vs. control mice (Ji et al., 2012b). A different study showed an attenuation of blood pressure and renal inflammation in Dahl salt-sensitive rats treated with a P2X7 antagonist (Ji et al., 2012a), suggesting that P2X7 is involved in hypertension and renal injury, potentially via an inflammatory mechanism.

### Do P2X4 and P2X7 interact in the kidney?

**Figure 2:**
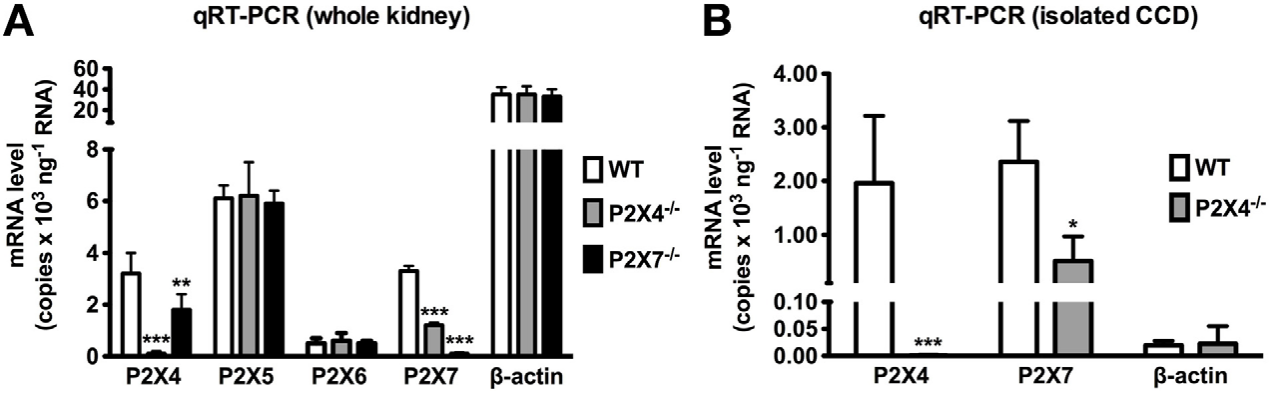
**(A)** P2X4, P2X5, P2X6, P2X7 and β-actin mRNA levels in the whole kidney from wild-type (WT), P2X4 null (P2X4^−/−^) and P2X7 null (P2X7^−/−^) mice. P2X4, 5, 6 and 7 mRNA was readily detected in WT kidney. P2X4 and P2X7 mRNA levels were > 100-fold lower in P2X4^−/−^ and P2X7^−/−^ mice, respectively (*n* = 3). P2X7 mRNA was significantly reduced in P2X4^−/−^ mice (*p* < 0.003; *n* = 6) and P2X4 mRNA was significantly reduced in P2X7^−/−^ mice (*p* < 0.03; *n* = 6). No significant difference was found in P2X5, P2X6 or β-actin between WT and either P2X4^−/−^ or P2X7^−/−^ mice. **(B)** P2X4 and P2X7 mRNA expression in microdissected cortical collecting ducts (CCD) from P2X4^−/−^ mice. P2X7 mRNA was significantly reduced in the CCDs of P2X4^−/−^ mice (*p* < 0.05; *n* = 3). ^*^P < 0.05; ^**^P < 0.03; ^***^P < 0.003.

Are the proposed interactions between P2X4 and P2X7 relevant in the kidney? Preliminary data from our laboratory suggests that genetic ablation of one may influence the renal expression of the other (Figure 2). mRNA measured in microdissected CDs and protein extracted from whole kidneys of P2X4^−/−^ mice revealed that levels of P2X7 were significantly reduced in P2X4^−/−^ mice compared with wild-type controls. P2X4 protein expression was also reduced in P2X7^−/−^ mice. The P2X4 protein decrease observed in P2X7^−/−^ mice was confined to the membrane fraction, whereas the P2X7 protein decrease in P2X4^−/−^ mice was seen in both the membrane and cytosolic fractions. These data suggest cross-talk between P2X4 and P2X7 subunits in the kidney, and certainly in the CD. The nature of the cross-talk between P2X4 and P2X7 observed in the kidney could be one of protein-protein interaction (i.e., a heterotrimeric assembly of P2X4 and P2X7 subunits) or of separate homotrimeric receptor coupling. Although this was not elucidated in this study, previous studies on other native and transfected systems favor the latter mechanism. Will these proposed physical interactions prove to have any functional relevance? They seem to in the immune system (Kawano et al., 2012a,b) and a similar story might hold in the kidney too.

## Discussion and future directions

The existence of physically associated P2X subunits, whether as heterotrimers or interacting homotrimers, implies a complexity to the purinergic signaling system that may serve to adjust the regulation of physiological processes and account for unexpected pharmacological characteristics. Certainly, the altered functional and pharmacological characteristics of P2X4 and P2X7 when they are present together in native tissue and co-expressed in heterologous systems, and of P2X7 when the expression of P2X4 is altered, suggest that the observed interactions are functionally relevant. Moreover, our own findings that the protein levels of each receptor are altered when one is genetically ablated, as well as P2X7 RNA expression in P2X4^−/−^ mice, is consistent with P2X4 and P2X7 functionally interacting in the kidney. However, evidence for functionally relevant P2X4 and P2X7 interactions in the kidney is circumstantial. The generation of a transgenic model that is null for both P2X4 and P2X7 would be useful in revealing the functional interdependence of these receptors, and such a model would also negate the potential of alterations in the expression of one receptor to compensate for loss of the other.

### Conflict of interest statement

The authors declare that the research was conducted in the absence of any commercial or financial relationships that could be construed as a potential conflict of interest.
